# Research on the Variable-Temperature Cracking Mechanism of CRTS I Type Double-Block Ballastless Track on a Bridge

**DOI:** 10.3390/ma15030770

**Published:** 2022-01-20

**Authors:** Zhiping Zeng, Guangzhao Peng, Weidong Wang, Xiangdong Huang, Shiwen Shen, Abdulmumin Ahmed Shuaibu, Xiaobai Meng

**Affiliations:** 1 School of Civil Engineering, Central South University, Changsha 410075, China; 203160@csu.edu.cn (Z.Z.); csupgz@csu.edu.cn (G.P.); wd1997@csu.edu.cn (W.W.); ShiwenShen@csu.edu.cn (S.S.); abdulshub4u@csu.edu.cn (A.A.S.); 204801060@csu.edu.cn (X.M.); 2 Ministry of Education Key Laboratory of Engineering Structures of Heavy Haul Railway, Central South University, Changsha 410075, China; 3 Department of Civil Engineering, Faculty of Engineering, Ahmadu Bello University, Zaria 800242, Nigeria

**Keywords:** solar radiation, temperature field, cracking mechanism, CRTS I TDBBT, sequential thermal stress coupled models

## Abstract

The CRTS I type double-block ballastless track (CRTS I TDBBT) has the advantages of convenient construction and low cost, but it has low crack resistance and the temperature field distribution of the railway on the bridge is uneven and frequently changes, so it is necessary to study the mechanical properties of the CRTS I TDBBT under the load of a temperature field. The temperature field model of the CRTS I TDBBT on the bridge is established by finite element software, the real-time temperature field of the track bed slab is brought into the coupled model as a load, and the variation laws of the temperature stress of the CRTS I TDBBT under different schemes are compared. The temperature gradient in the CRTS I TDBBT track bed slab has the largest fluctuation range, and the positive and negative temperature gradient range can reach 93.34 °C. For the temperature longitudinal stress around the sleeper block of the track bed slab, the edge is the largest; the temperature longitudinal stress is reduced by at most 5.27% after the anti-cracking diagonal bars are added. When the expansion joint is added, the temperature stress can be reduced by up to 80.29%. The fluctuation range of the temperature gradient of the track bed is basically consistent with the fluctuation range of the local air temperature. The huge temperature difference leads to the occurrence of cracks in the track structure, and cracks are more likely to occur at the corners of the sleeper block. The addition of both anti-crack diagonal bars and expansion joints has an anti-crack effect, but the effect of adding expansion joints is better.

## 1. Introduction

**Figure 1:**
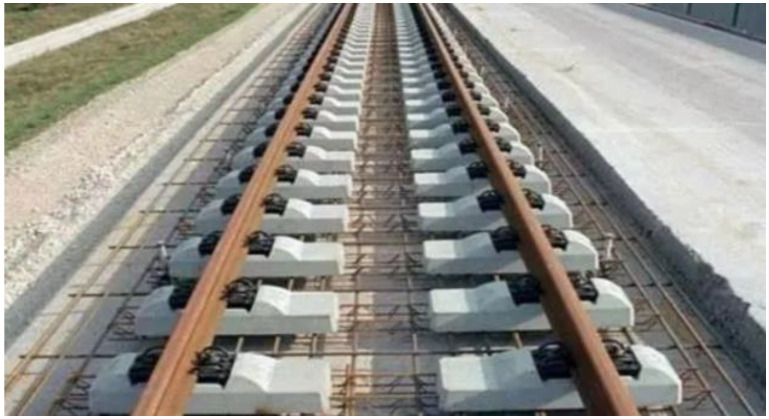
CRTS I TDBBT.

**Figure 2:**
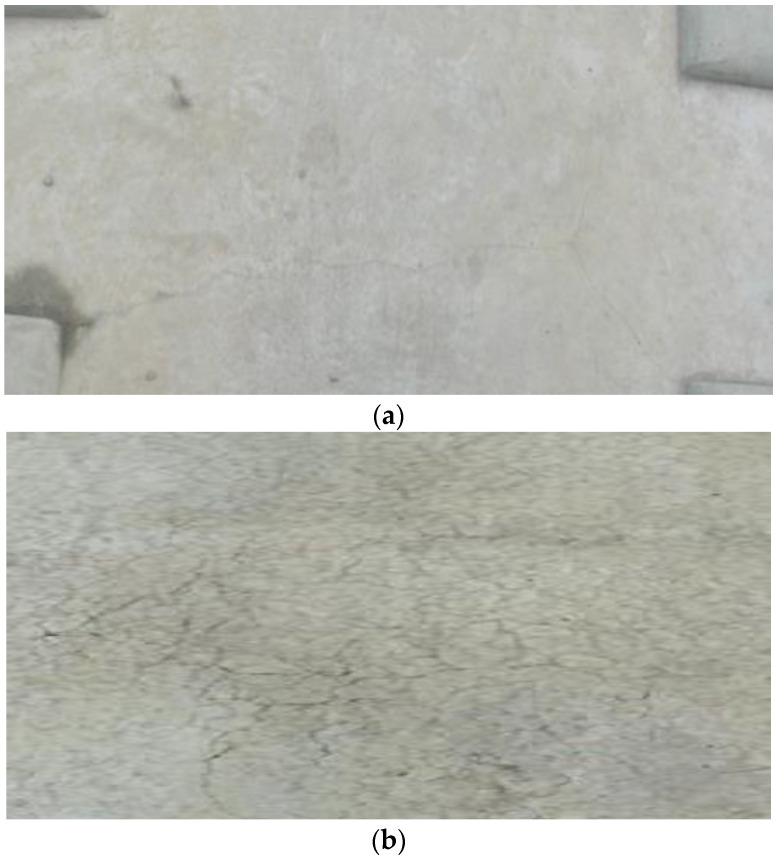
Double-block ballastless track issues. (**a**) The corner crack in the edge of the track sleeper. (**b**) The map crack in the middle of the slab.

During the operation of high-speed railway, the ballastless track develops cracks, upwarps, and faces other issues under the combined effects of temperature load and rain erosion [1,2]. This not only seriously affects its service performance and leads to a high maintenance cost but also affects its strength, fatigue performance, and stability [3,4]. Since the sleeper of a CRTS I type double-block ballastless track (CRTS I TDBBT) is prefabricated in the factory, and the track slab is cast on site, there is contact action between new and old concrete and uneven distribution of the temperature gradient occurs, resulting in surface cracks and damages in the contact zone between the double-block sleeper and the track slab [5,6]. The CRTS I TDBBT is shown in Figure 1. The corner crack in the track slab has three forms [7]: the corner crack at the edge of the track sleeper, the map crack in the middle of the slab, and the lateral deep crack, as shown in Figure 2.

Because the ballastless track slab and the base slab are greatly affected by temperature, it is difficult to avoid concrete cracking when the temperature changes [8,9]. On-site investigation found that the cracking phenomenon of the track slab depends on the on-site use [10]. Cracking will cause a sudden increase in the stress of the steel bar, and rainwater and air will penetrate into the steel bar inside the concrete, which will cause the steel bar to corrode [11]. The durability of the ballastless track affects the stability of the track structure and the stability and safety of high-speed driving [12,13]. Therefore, it is necessary to conduct a systematic study on the causes, characteristics, and effects of cracks when the temperature of the ballastless track changes, which could provide theoretical support for crack control and maintenance management.

Many institutions have launched a series of studies on the temperature cracks of the track slab. Bin et al. [14] established a finite element model of the CRTS I TDBBT structure on the subgrade and studied the mechanics and crack characteristics of the CRTS I TDBBT on the subgrade under temperature and shrinkage loads. Zhenmin et al. [15,16] analyzed the cracks of the double-block cast-in-situ concrete track slab on the test section of the Sui-Yu line and proposed specific measures to prevent and control the cracks of the CRTS I TDBBT.

Based on the principle of heat transfer, Fushan et al. [17] established the CRTS II track three-dimensional temperature field finite element analysis model and studied the time-varying law of the temperature field distribution inside the track slab, as well as the solar radiation, the wind speed, and the heat transfer of the mortar layer. Factors such as performance and environmental change speed have an influence on the temperature field of the track slab. Xueyi et al. [18] analyzed the relationship between the temperature of the track slab and environmental factors based on the principle of thermodynamics; they established a simple calculation method for the temperature of the track slab and analyzed the temperature characteristics of the track slab under the extreme weather in Chengdu.

The theoretical analysis of all the above temperature fields involves basically the temperature load under specific conditions of the ballastless track or the relationship between the track temperature field and meteorological factors. It can be seen that most of the existing research objects are static and the research content is relatively simple, making it difficult to reflect the change law of the track slab under the changing temperature field.

Since the CRTS I TDBBT adopts cast-in-place construction without prestressed steel bars and has low crack resistance, while the temperature field distribution of the railway on the bridge is uneven and frequently changes, it is necessary to study the mechanical properties of the CRTS I TDBBT under a temperature field load to guide related engineering construction.

## 2. The Establishment of a Finite Element Model

### 2.1. Basic Dimensions and Parameter Settings of the Model

The standard size of the concrete track slab in the bridge section is selected to be 6400 mm long, 2800 mm wide, and 260 mm thick. Each track slab on the bridge is provided with two restrict bosses protruding in the direction of the support. The restrict boss is quadrangular in the height direction, the inclination is 1:10, and the dimensions of the upper and lower surfaces are 1022 mm × 700 mm and 1000 mm × 678 mm, respectively, with a height of 110 mm. The concrete support on the bridge adopts a block structure, and the length and width of the support are the same as those of the track slab, with a height of 210 mm. Two grooves are provided on each support of the bridge to match the restrict boss of the track slab.

**Figure 3:**
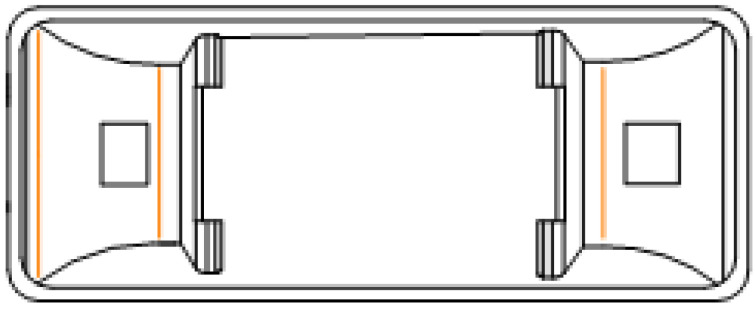
Double-block sleeper after processing optimization.

The track slab on the subgrade is continuously poured, the average thickness of the track slab is 260 mm, and the width is 2800 mm. The track slab is made of cast-in-situ C40 reinforced concrete. The support layer has a width of 3400 mm and a thickness of 300 mm. Since expansion joints have little effect on temperature and stress coupling, expansion joints are no longer considered in the modeling process. Both the track slab and the supporting layer are provided with a lateral slope, which does not have much influence on the deformation of the track structure, so it is simplified. To subsequently explore the contact stress between the double-piece sleeper and the track slab, the double-block sleeper in this research adopts the rounded corner treatment to optimize the sleeper block, as shown in Figure 3. To facilitate the division and calculation of the unit, this research simplifies the upper part of the sleeper block.

**Figure 4:**
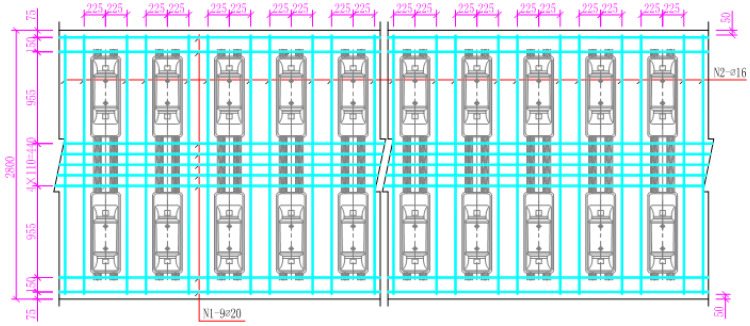
Arrangement of steel bars on the upper and lower floors of the track bed.

Nine Φ20 longitudinal steel bars are arranged on the upper and lower floors of the track bed, and two Φ16 lateral steel bars are arranged between every two sleepers. The concrete protection layer is designed to be 50 mm thick, as shown in Figure 4. (The physical quantities in the figure are all millimetres).

**Figure 5:**
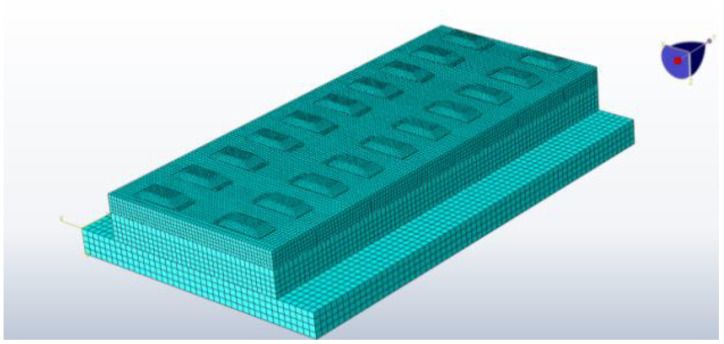
Three-dimensional geometric model of the CRTS I TDBBT structure.

**Figure 6:**
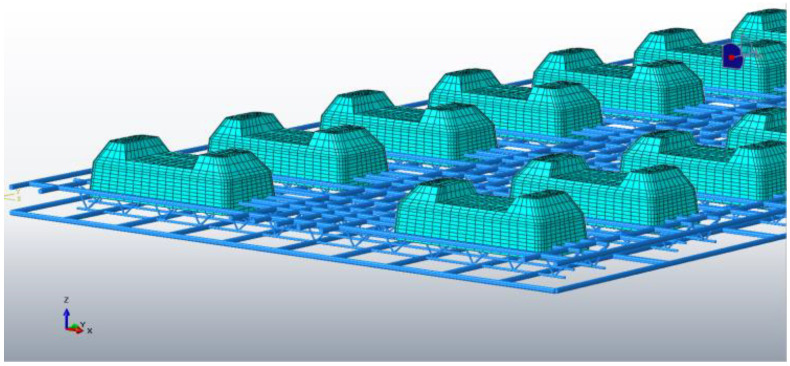
Line element 3D model of internal reinforcement of the CRTS I TDBBT structure.

Based on the above geometric conditions, a three-dimensional geometric model of the CRTS I TDBBT structure is established, as shown in Figure 5, and the reinforcement inside the track slab is simulated using line elements, as shown in Figure 6.

### 2.2. Material Parameters and Working Boundary Conditions

The structure and main dimensions of the CRTS I TDBBT are as described in Section 2.1. The sleepers adopt a C60 concrete structure, the track slab adopts a C40 reinforced concrete structure, and the supporting layer adopts a C15 concrete structure. The specific physical parameters of each structure are shown in Table 1.

The boundary conditions are convective heat transfer conditions and radiation heat transfer conditions at atmospheric temperature, the load is the solar radiation heat flux density, the initial temperature is set at 30 °C, the surface-to-surface contact is set as a bonded contact, and the element adopts a heat transfer three-dimensional hexahedral element. The temperature field calculation model of the CRTS I TDBBT structure is established by finite element software.

Considering the actual operation of the CRTS I TDBBT, the external load conditions are divided into three types, namely temperature load conditions, external constraints, and gravity fields. The temperature load is the result of the pure thermal analysis of the CRTS I TDBBT. The external restriction is to constrain the longitudinal direction of the longitudinal sides of the bridge and the support, that is, U2 = UR1 = UR3 = 0, and completely constrain the bottom surface of the bridge, that is, U1 = U2 = U3 = UR1 = UR2 = UR3 = 0. The gravity field is the self-weight of the CRTS I TDBBT. The binding contact way is adopted among the support, the track slab, and the sleeper, and the contact way between the reinforcement and the track slab is embedded. After calculation, the results of sequential thermal stress analysis of the CRTS I TDBBT can be obtained.

### 2.3. Simulation Method of the Temperature Field Load

The temperature field of the track structure is a nonlinear instantaneous change under complex conditions. To obtain the temperature field distribution of the CRTS I TDBBT structure under natural conditions, the finite element software is used to simulate the geometric, physical, and load conditions of the track slab and the temperature field model is analyzed.

The operation results of the temperature field pure thermal model are brought into the thermal stress coupled model, and a sequential thermal stress analysis is carried out [19,20,21]. The temperature stress distribution of the CRTS I TDBBT can be obtained and further analyzed.

**Figure 7:**
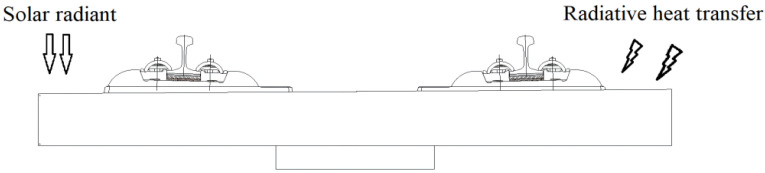
Schematic diagram of the temperature field model of the CRTS I TDBBT structure.

The temperature field analysis model of the CRTS I TDBBT, which essentially consists of a heat conduction analysis under the initial conditions of track and boundary conditions [22,23,24], is established. The main effects of the temperature field change on the CRTS I TDBBT structure results from solar radiation, radiative heat transfer, and convective heat transfer [25], as shown in Figure 7.

Therefore, the temperature field analysis model of the CRTS I TDBBT can be obtained [26], as shown in Equation (1).

(1) $${-\lambda \frac{\partial T}{\partial n}|}_{s}=Q+q_{c}+q_{r}$$

where *Q* is the solar radiant heat flux, in W/m^2^; *q_c_* is the radiative heat transfer heat flux, in W/m^2^; and *q_r_* is the convective heat transfer heat flux, in W/m^2^.

The boundary conditions mainly reflect the degree of interrelationship between the object under study and the surrounding environment. The convective heat transfer and the radiative heat transfer coexist on the surface of the track, which is a complex case of the third type of boundary conditions in heat transfer [27] and can be expressed by Equation (2):

(2) $${-\lambda \frac{\partial T}{\partial n}|}_{s}=\alpha_{s}Q(\tau )+h(T_{s}-T_{\infty })+h_{r}(T_{s}-T_{\infty })$$

where $\alpha_{s}$ is the radiation absorption rate of the track surface; *h* is the convective heat transfer coefficient of the track surface; $h_{r}$ is the radiative heat transfer coefficient of the track surface; and $T_{s}$ and $T_{\infty }$ are the air temperature and the surface temperature of the track, respectively.

The double-block sleeper is in close contact with the track slab, and the track slab is in close contact with the support, which satisfies the fourth type of boundary condition of heat transfer [28], which is, the contact surface temperature is equal and the heat flux density is equal. Therefore, the contact setting is made by face-to-face bonding.

The dynamic temperature is fitted by a double sine function that reflects the law of temperature change on a sunny day [29], as shown in Equation (3).

(3) $$T_{a}=E(T)+\Delta T\left\{\begin{array}{l} 0.96\sin\left[\omega \left(\tau -\tau_{0}\right)\right]\\ +0.146\sin\left[2\omega \left(\tau -\tau_{0}\right)\right] \end{array}\right\}$$

where *E(T)* is the daily average temperature; Δ*T* is the difference between the daily maximum temperature and the daily minimum temperature; τ is the time calculated in hours and is set to 0 at 6 am; $\tau_{0}$ is a constant time, equal to 3; and ω is the frequency and ω = 2π/24.

### 2.4. Model Reliability Verification

**Figure 8:**
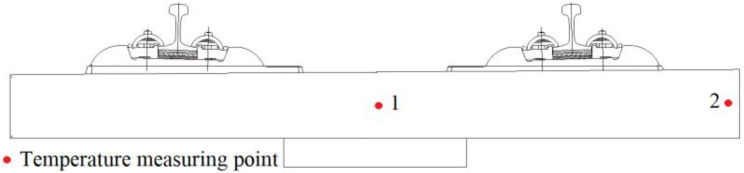
Layout of measuring points.

To verify the reliability of the model, the test data of the test section on the roadbed of the Qingrong Intercity Railway linking Taojiakuang Tunnel and Wangjiazhuang Bridge in Shandong Province, China, are used. A comparative study is carried out between the measured model and the theoretical model data, and two measuring points in the middle of the axis of the ballast bed are selected for model verification, as shown in Figure 8.

**Figure 9:**
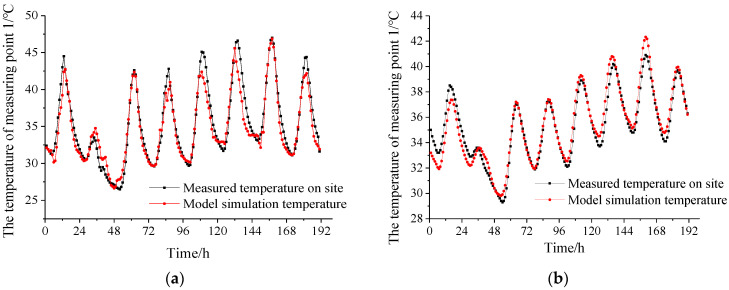
Comparison of temperature test data and model data. (**a**) Temperature measuring point 1. (**b**) Temperature measuring point 2.

The temperature field model of the CRTS I TDBBT is established according to the conditions mentioned above, and the temperature distribution is obtained by finite element software calculation. The test data from 14 to 21 July is used to compare with the calculation results of the corresponding points in the model, as shown in Figure 9.

According to Figure 9, the temperature test data of each measuring point and the model data can be well matched in terms of temperature curve change period, vibration amplitude, and numerical value. There is an error between the test temperature of some measuring points and the model temperature at individual moments. Using the improved Euclidean average distance formula with the introduction of the balance offset factor [30], a similarity analysis of the two groups of time series data of the test and the model is carried out, as shown in Equation (4).

(4) $$D(A,B)=\sqrt{\sum_{i=1}^{n}{\left|a_{i}-b_{i}-m\right|}^{2}},\;m=\sum_{i=1}^{n}\left(a_{i}-b_{i}\right)/n$$

In the equation, *D(A,B)* is the Euclidean distance, and the smaller *D(A,B)* is, the closer the two sets of time series data are; [*a_i_*] is the test temperature time series data; [*b_i_*] is the temperature time series data calculated by the model; *m* is the balance offset factor; and *n* is the number of data.

The calculated Euclidean average distances of measuring point 1 and measuring point 2 are 0.1314 and 0.3597 °C, respectively, which are both less than 0.5 °C. It can be seen that the CRTS I TDBBT temperature field model has strong adaptability to the track structure simulation under natural conditions.

## 3. Analysis of Results of the Pure Thermal Model of the CRTS I TDBBT on the Bridge

### 3.1. Selection of Temperature Load Parameters and Optimization Scheme

The main purpose of this study is to analyze the feasibility of laying the CRTS I TDBBT on the bridge railway, so the temperature change in Huaian County of Beijing-Zhangjiakou Railway is taken as an example. Huaian County is located in the basin between the mountains. The historical daily temperature difference is large, and the cracks in the track slab are more frequent. Therefore, a feasibility study for laying of the CRTS I TDBBT on the bridge in this section is explored.

**Figure 10:**
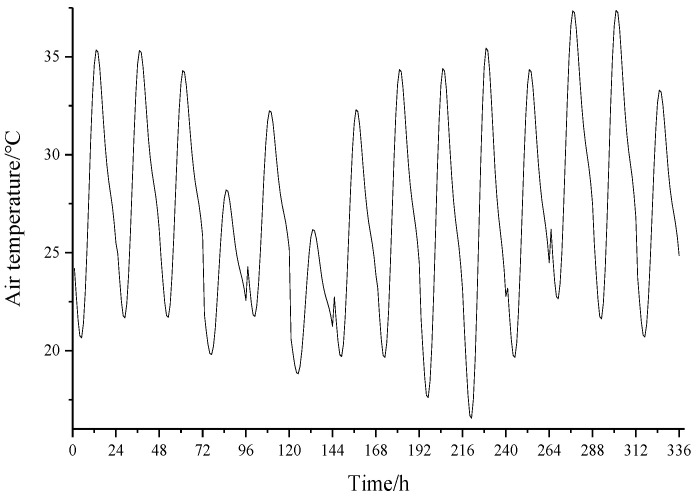
Time-varying temperature diagram of the Huaian County section.

The geographic latitude of Huaian County is 40.4° North. According to the railway line alignment, the normal directions of the two sides of the track slab are −30° and 150° from the south direction. The normal directions of the vertical plane of the double-block sleeper are −30°, 60°, 150°, and −120° from the south direction. The local atmospheric transparency coefficient in July is recorded as 0.66. Therefore, the time-varying temperature of Huaian County from 1 to 14 July can be fitted, as shown in Figure 10. Therefore, select and import the Creat Predefined Field module in the finite element software and each element node in the model will load the temperature values at each moment in the two weeks from 1 to 14 July, in turn, to simulate the temperature change in the CRTS I TDBBT structure in actual operating conditions.

To further explore the influence mechanism of the temperature load on the CRTS I TDBBT on the bridge and provide a solution to the damage caused, on the basis of the original scheme, two optimal design schemes are proposed for comparison with the original scheme. Therefore, the following three calculation conditions are proposed:

Working condition 1 (original scheme): Normal construction and reinforcement of the track slab.

Working condition 2 (optimal design Scheme 1): On the basis of normal construction and reinforcement situation, 10 sets of Φ12 anti-cracking reinforcements are added to the upper layer on the track slab. With 8 reinforcements in each set, each anti-cracking reinforcement is 40 mm long and is distributed in the four corners of the sleeper block.

**Figure 11:**
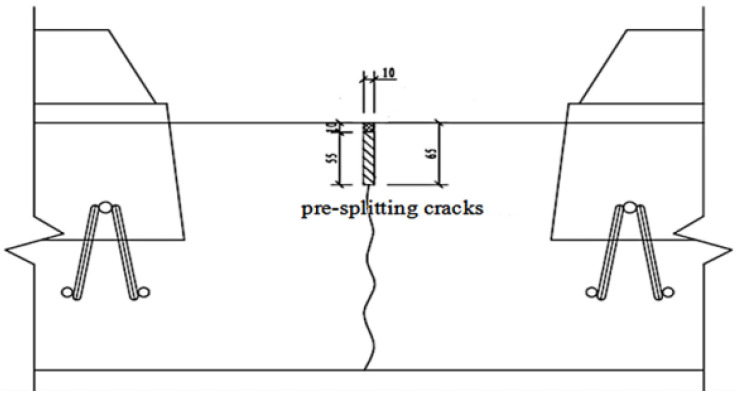
The track slab with increased expansion joints.

Working condition 3 (optimal design Scheme 2): Adding expansion joints to the track slab, the pre-splitting cracks of the track slab is 10 mm wide and 65 mm deep, as shown in Figure 11.

### 3.2. Analysis of the Vertical Temperature Gradient Distribution

The temperature field model for pure thermal analysis is established based on the numerical calculation model considering the effects of solar radiative heat, convective heat transfer, radiative heat transfer, and the ambient temperature of the CRTS I TDBBT on the bridge in the Huaian County section of the Beijing–Zhangjiakou Railway.

**Figure 12:**
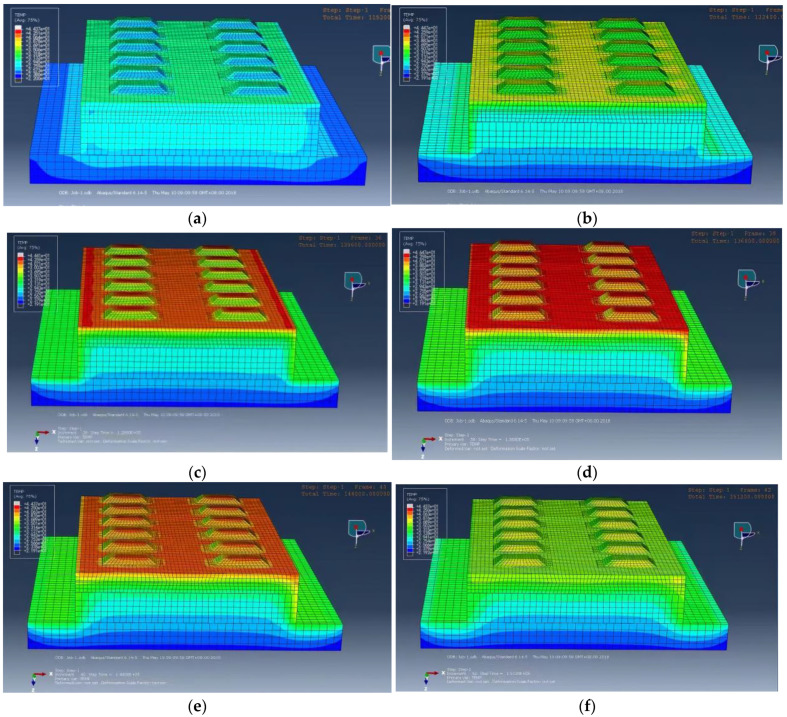
Distribution of the temperature field variation of the CRTS I TDBBT in 1 day. (**a**) 7 h, (**b**) 11 h, (**c**) 13 h, (**d**) 15 h, (**e**) 17 h, and (**f**) 20 h.

The CRTS I TDBBT model on the bridge is broken laterally along the longitudinal middle line, and the temperature field distribution of the CRTS I TDBBT at various times in 1 day can be obtained, as shown in Figure 12.

**Figure 13:**
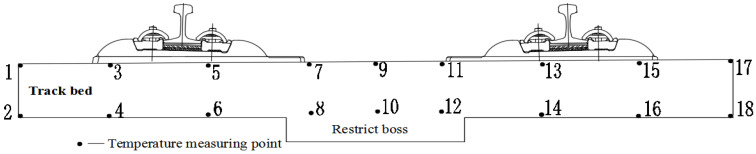
Track slab temperature measurement point layout.

As observed from Figure 12, the external temperature of the track slab is greatly affected by solar radiation and the surrounding atmosphere temperature. The temperature variation is more obvious and changes regularly every day, while the internal temperature of the track slab is less affected by the external environment because the concrete has a certain thermal insulation. Therefore, the positive and negative temperature gradients of the track slab are alternately changed. To further explore the variational distribution, 18 points on the lateral medial axis of the track slab are selected and a total of 9 vertical temperature gradients are used, as shown in Figure 13.

**Figure 14:**
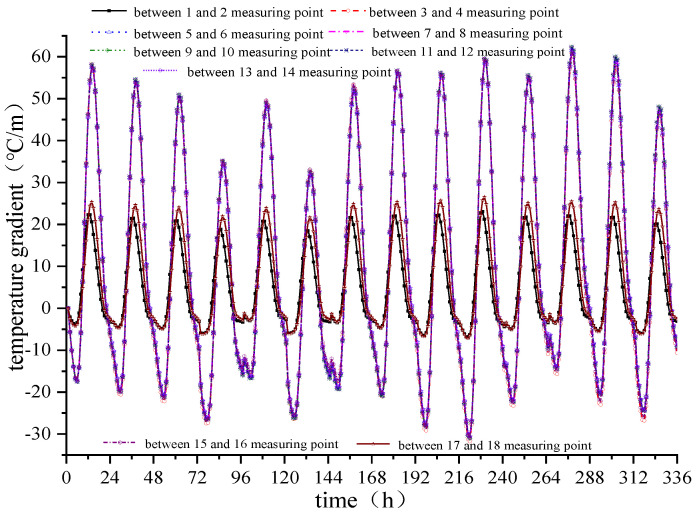
Time-dependent deformation of temperature at each measuring point of the track slab.

The temperature measurement point data is extracted from the model and the corresponding nine vertical temperature gradients are obtained, as shown in Figure 14. The vertical temperature gradient between the first and second measuring points is similar to the vertical temperature gradient between the 17th and 18th measuring points and changes periodically between +25 and −5 °C/m. When the lateral plate is close, the temperature gradient increases rapidly. When the middle of the lateral track slab is reached, that is, between the 9th and 10th measuring points, the temperature gradient changes the most and changes periodically between +60 and −30 °C. This is consistent with the maximum positive and negative temperature gradients of 90 °C/m and −45 °C/m, respectively, in China’s ballastless track slab specification.

### 3.3. Analysis of the Time-Dependent Deformation of the Vertical Temperature Gradient of the Track Slab

**Figure 15:**
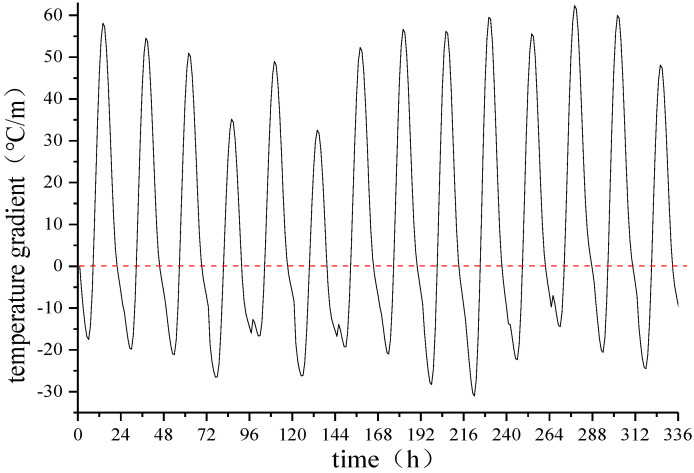
Time-dependent deformation of the temperature gradient in the middle of the track slab.

Because the vertical temperature gradient of the track slab between the 9th and 10th measuring points is the largest, the temperature gradient between the two measuring points is taken as a critical point and the time-dependent deformation of the track slab subject to the temperature gradient of the track slab is further explored, as shown in Figure 15.

As observed from Figure 15, the temperature gradient at the lateral centerline of the track slab changes regularly on a day-to-day cycle, with a positive temperature gradient from 8:00 to 20:00 hours and a negative temperature gradient at other times. At 13:00 hours every day, the maximum positive temperature gradient is reached, which is +62.32 °C. The maximum negative temperature gradient is reached at 5:00 hours every day, which is −31.02 °C, and the positive and negative temperature gradients can reach 93.34 °C.

## 4. Analysis of Results of the Sequential Thermal Stress Coupled Model of the CRTS I TDBBT on the Bridge

The sequential thermal stress coupled model of the CRTS I TDBBT on the bridge is analyzed, and the corresponding temperature thermal stress data are obtained and compared under different working conditions.

### 4.1. Analysis of the Issues of the Track Slab with Normal Reinforcement under a Temperature Load

**Figure 16:**
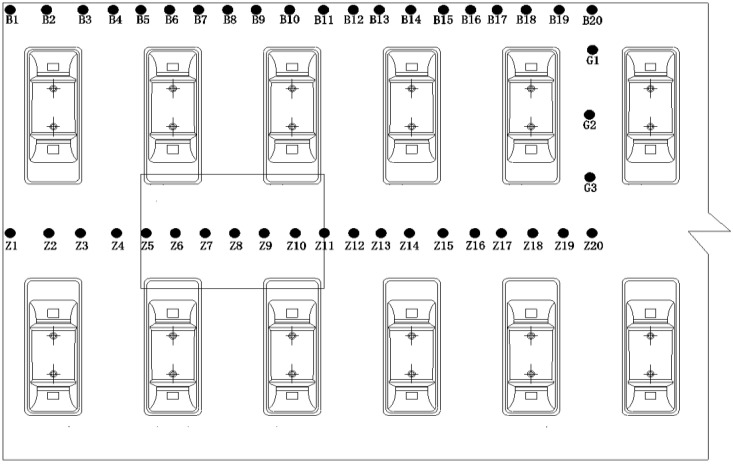
Temperature stress measuring point layout of the track slab.

To further explore the temperature stress distribution and crack forming mechanism of the CRTS I TDBBT on the bridge, the temperature stress measuring points are arranged on the surface of the track slab, as shown in Figure 16. The arrangement is as follows: the longitudinal edge measuring points B1 to B20 of the track slab, the middle measuring points G1 to G3 between the sleeper blocks, the longitudinal middle measuring points Z1 to Z20 of the track slab, which makes 23 temperature stress measuring points in total.

**Figure 17:**
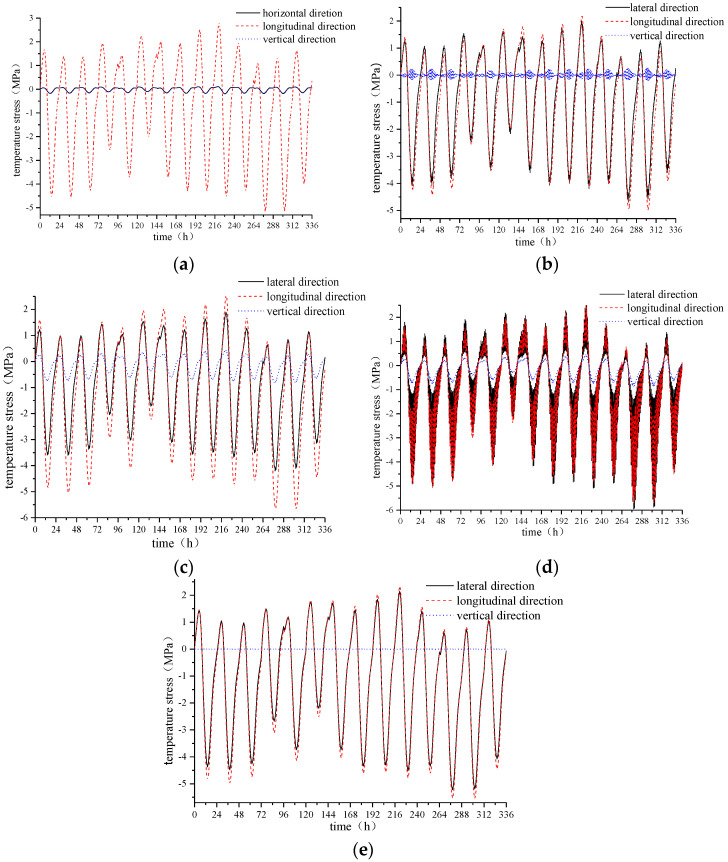
Time-dependent deformation of temperature stress in three directions of each measuring point on the track slab. (**a**) B20 measuring point, (**b**) G1 measuring point, (**c**) G2 measuring point, (**d**) G3 measuring point, and (**e**) Z20 measuring point.

Five points, B20, G1, G2, G3, and Z20, are selected, and the vertical stress, the lateral stress, and the longitudinal stress of the five points are analyzed and compared, as shown in Figure 17.

The vertical temperature stress at each position is much smaller than the lateral and longitudinal temperature stresses. The lateral temperature stress at the B20 measurement point is much smaller than the longitudinal temperature stress, and the lateral temperature stress at the other measurement points is slightly smaller than the respective longitudinal temperature stress. Therefore, cracks are more likely to occur in the lateral centerline of the track slab and the sleeper, while 45° diagonal cracks are more likely to occur at the four corners of the sleeper, which can propagate laterally because the longitudinal temperature stress is greater than the lateral temperature stress. At the edge of the track slab, since the longitudinal temperature stress is much larger than the lateral and vertical temperature stresses, the vertical longitudinal crack is more likely to occur.

In addition, the lateral temperature stress change at each measuring point of the track slab is in the sequence J19 > Z20 > GZ14 > Z19 > B20. The longitudinal temperature stress change at each measuring point of the track slab is in the sequence J19 > Z19 > Z20 > B20 > GZ14. Therefore, since the lateral and longitudinal temperature stresses are the largest at the four corners where the sleeper and the track slab are in contact, the position is more likely to be obliquely cracked.

### 4.2. Longitudinal Temperature Load Distribution on the Surface of the Track Slab under Normal Reinforcement

**Figure 18:**
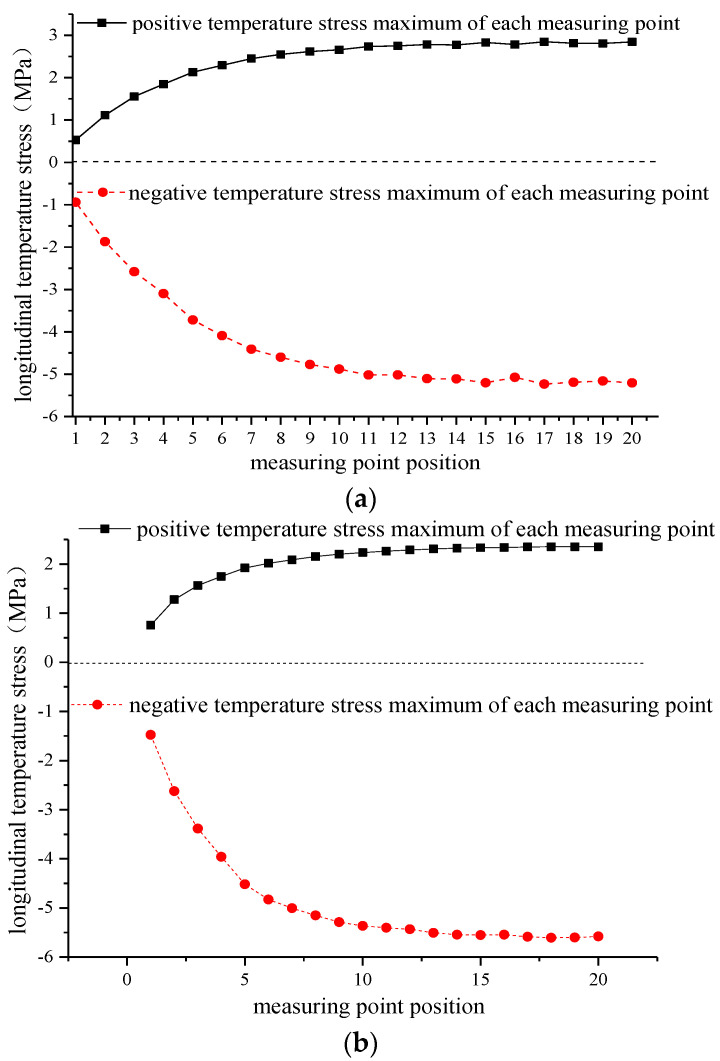
Variation in the longitudinal temperature stress of the track slab. (**a**) Measuring point at the edge of the track slab. (**b**) Measuring point in the middle of the track slab.

The longitudinal temperature gradient of each measuring point is selected for comparative analysis. The selected points are measuring points B1 to B20 at the lateral edge of the track slab and DZ1 to DZ20 in the lateral middle of the track slab. The maximum longitudinal positive temperature gradient and the maximum longitudinal negative temperature gradient of each measuring point are selected to further investigate the longitudinal temperature stress distribution in the track slab, as shown in Figure 18. Among them, “temperature stress” refers to the stress value inside the structure caused by the structure under the action of the temperature load. When the temperature decreases, the object shrinks and the internal phases of the structure stretch each other and generate tension, which is called “positive temperature stress”. When the temperature increases, the object expands and the internal phases of the structure squeeze each other and generate pressure, which is called “negative temperature stress”.

As observed from Figure 18a above, for the longitudinal edge of the track slab, the absolute values of longitudinal positive and negative temperature stresses of the measuring point B1 are the smallest, 0.528 and −0.940 MPa, respectively. Toward the middle of the track slab, a gradual increase in longitudinal positive and negative temperature stresses is observed. At the measuring point B20, the longitudinal positive and negative temperature stresses are the largest, with respective values of 2.843 and −5.205 MPa. The longitudinal maximum positive temperature stress is increased by 5.4 times, while the longitudinal maximum negative temperature stress is increased by 5.5 times when compared with the B1 measuring point.

In addition, it can be observed from Figure 18b that the longitudinal positive and negative temperature stresses of the measuring point DZ1 are the smallest, 0.755 MPa and −1.476 MPa, respectively. With the extension to the middle of the track slab, the longitudinal positive and negative temperature stresses gradually increase. At the measuring point DZ20, the longitudinal positive and negative temperature stresses are the largest, with respective values of 2.354 MPa and −5.779 MPa. While the longitudinal maximum positive temperature stress increases by 3.1 times, the longitudinal maximum negative temperature stress increases by 3.8 times when compared with the DZ1 measuring point. In summary, from the longitudinal edge of the track slab to the middle, the longitudinal temperature stress of each measuring point gradually increases, and at the longitudinal centerline of the track slab, the longitudinal temperature stress reaches the maximum value, which is more likely to cause cracking due to the temperature stress of the track slab.

### 4.3. Longitudinal Temperature Load Distribution between Layers of the Track Slab under Normal Reinforcement

**Figure 19:**
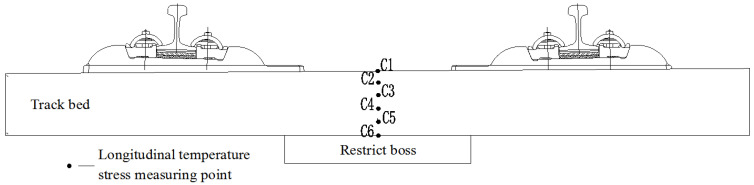
Temperature stress measuring point layout of the cross section in the middle of the track slab.

**Figure 20:**
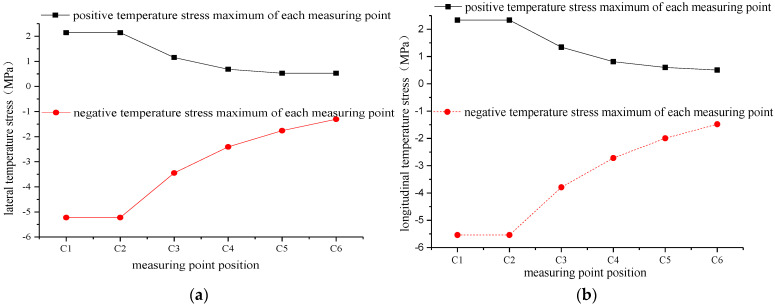
Variation in temperature stress extremes at each measuring point between the layers of the track slab. (**a**) Lateral temperature stress. (**b**) Longitudinal temperature stress.

It can be observed from the discussion above that the longitudinal temperature stress in the track slab is the maximum value of the overall temperature stress, which has a great influence on the surface cracking of the track slab. For the change of the temperature stress between the layers of the track slab, the longitudinal middle cross section of the track slab is selected. In the cross section, the track slab is divided into five layers, each layer is 52 mm thick, and the temperature stress measurement points are arranged in the lateral middle of each layer. As shown in Figure 19, the maximum positive and negative temperature stresses of each measuring point are selected to further explore the vertical distribution of the temperature stress at the measuring point in each layer, as shown in Figure 20.

From Figure 20, it can be seen that the lateral and longitudinal temperature stresses of each measuring point are gradually reduced on extending downward from the surface of the track slab. The lateral temperature stress decreases from −5.224–2.144 MPa to −1.307–0.526 MPa. The positive and negative temperature stresses are reduced by 75.5%, and the longitudinal temperature stress range is decreased from −5.541–2.373 MPa to −1.481–0.505 MPa. In other words, the positive temperature stress decreases by 78.4% and the negative temperature stress decreases by 73.3%. Therefore, the longitudinal and lateral temperature stresses are greater close to the surface of the track slab and their influence on the track slab is more significant.

### 4.4. Analysis of Temperature Stress Change after Adding Anti-Cracking Reinforcement

**Figure 21:**
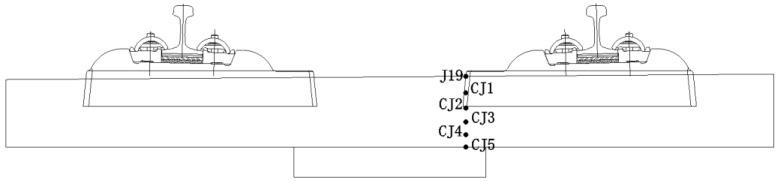
Temperature stress measuring point layout of the cross section of the contact between the longitudinal middle of the track slab and the sleeper.

**Figure 22:**
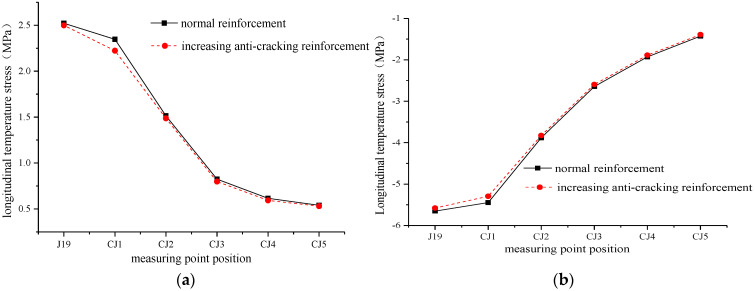
Comparison of positive and negative extremes of longitudinal temperature stress at each measuring point under two working conditions. (**a**) Positive temperature stress. (**b**) Negative temperature stress.

Because the contact between the longitudinal middle of the track slab and the sleeper is the position where the lateral temperature stress and the longitudinal stress are the largest, the measuring point J19 at the edges of the fifth sleeper in Figure 4 is selected and divided into five layers in the vertical direction, CJ1, CJ2, CJ3, CJ4, and CJ5, as shown in Figure 21. This is to further explore the effect of increasing the anti-cracking reinforcement of the track slab. The positive and negative extremes of the longitudinal temperature stress of the five measuring points under the two working conditions are also compared, as shown in Figure 22.

From Figure 22, it can be seen that after increasing the anti-cracking reinforcement, the longitudinal temperature stress of each measuring point is reduced and the reduction in the longitudinal temperature stress is more obvious because CJ1 is closer to the increased reinforcement. The positive temperature stress reduction is the highest at point CJ1, which is reduced by 5.27%, and the decrease is the lowest at point J19, which is reduced by 0.92%. The negative temperature stress reduction is the highest at point CJ1, which is 2.79%, and the decrease is the lowest at point J19, which is 1.32%. Therefore, increasing the anti-cracking reinforcement can appropriately reduce the longitudinal stress of each layer of the track slab.

### 4.5. Analysis of Temperature Stress Change after Adding Expansion Joints

**Figure 23:**
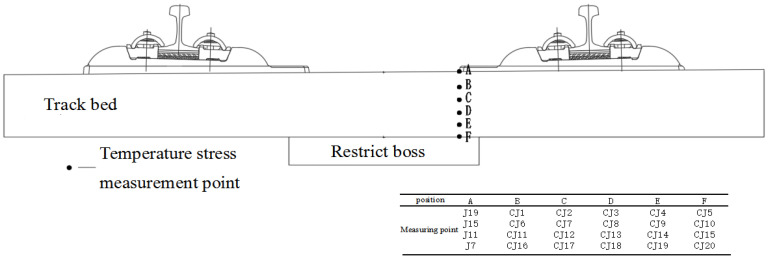
Temperature stress point layout at the edge and corners of the sleeper.

**Figure 24:**
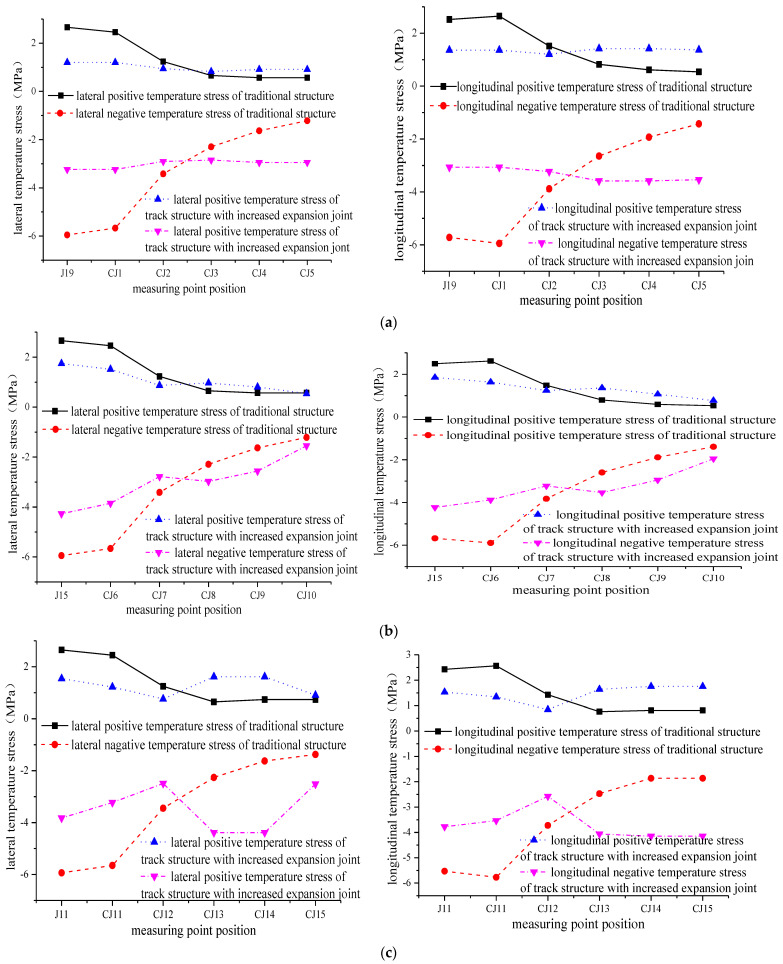

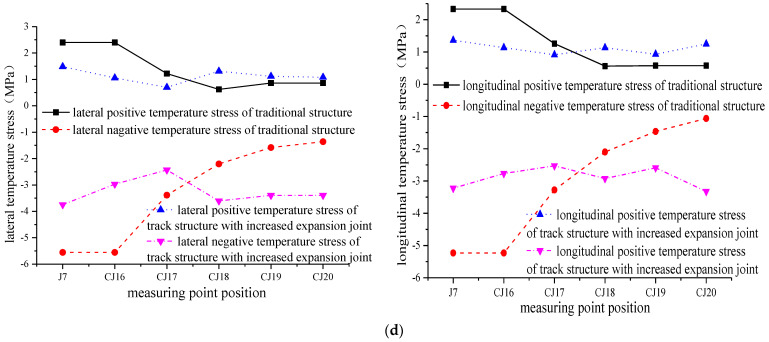
Comparison of positive and negative temperature stress extremes at each measuring point under two working conditions. (**a**) The contact position between the track slab and the edge and corners of the fifth sleeper block. **Left**: Lateral temperature stress. **Right**: Longitudinal temperature stress. (**b**) The contact position between the track slab and the edge and corners of the fourth sleeper block. **Left**: Lateral temperature stress. **Right**: Longitudinal temperature stress. (**c**) The contact position between the track slab and the edge and corners of the third sleeper block. **Left**: Lateral temperature stress. **Right**: Longitudinal temperature stress. (**d**) The contact position between the track slab and the edge and corners of the second sleeper block. **Left**: Lateral temperature stress. **Right**: Longitudinal temperature stress.

Because the contact between the longitudinal middle of the track slab and the sleeper is the position where the lateral temperature stress and the longitudinal stress are the largest, points J7, J11, J15, and J19 are selected from the inner edge of the second to fourth sleeper, which is in contact with the track slab surface. As shown in Figure 23, each measuring point is divided into five layers in the vertical direction, and each layer takes five measuring points. A total of 24 measuring points are taken to further explore the effect of increasing the expansion joint on the track slab. The positive and negative extremes of the longitudinal temperature stress of the 24 measuring points are compared under two working conditions, as shown in Figure 24.

According to Figure 24, it can be seen that in the contact position between the track slab and the edge and corners of the sleeper, the lateral and longitudinal temperature stresses of the first three measuring points of the track with an increased expansion joint are much smaller than those of the traditional track from the surface to the bottom of the track slab. The lateral and longitudinal temperature stresses of the last three measuring points of the track with an increased expansion joint are larger than those of the traditional track. However, as a whole, the lateral temperature stress and the longitudinal temperature stress of the track with an increased expansion joint are smaller than those of the traditional track. Therefore, an increase in expansion joints is useful in preventing surface cracking of the track slab when subjected to thermal stresses.

To further obtain the excellent conditions of the two working conditions, 0 MPa is used as the temperature stress origin and the standard value of the third-order origin moment is used to further reflect the direction and extent of the lateral and longitudinal temperature stress data distribution relative to the origin. The standard value of the second-order origin moment is used to further reflect the degree of dispersion of the lateral and longitudinal temperature stress data distribution relative to the origin. After calculation, the standard value of the third-order origin moment of the temperature stress of the track on the traditional bridge is −3.104 MPa and the standard value of the second-order origin moment is 2.814 MPa. The third-order origin moment of the temperature stress of the track with an increased expansion joint is increased to −2.622 MPa, and the second-order origin moment standard value is 2.496 MPa. Therefore, it can be concluded that the third-order origin moment and the second-order origin moment of the temperature stress of the track after increasing the expansion joint are reduced, which indicates that the degrees of deviation and dispersion of the temperature stress are smaller than those of the traditional track. Thus, the resistance to temperature load is better than that of the traditional structure.

### 4.6. Comparison of Maximum Temperature Stress and Temperature Gradient

It can be seen from the above that the longitudinal temperature stress is much greater than the lateral temperature stress and the vertical temperature stress and all the maximum temperature stresses are the maximum longitudinal temperature stresses under the effect of the maximum positive temperature gradient of 62.32 °C and the maximum negative temperature gradient of −31.02 °C.

The standard value of the third-order origin moment of the temperature stress of the original design scheme is −3.104 MPa, and the standard value of the second-order origin moment is 2.814 MPa. The standard value of the third-order origin moment of the track bed slab temperature stress of the track structure with expansion joints is −2.622 MPa, and the standard value of the second-order origin moment is 2.496 MPa.

According to the “Code for design of concrete structures” [31], the cracks in the track slab are mainly caused by tensile stress. The standard tensile strength of C50 concrete is 2.65 MPa. If there is no expansion joint, the positive temperature stress will exceed the tensile strength of C50 concrete and cause cracking, and after adding expansion joints, the calculated maximum positive temperature stress is less than the tensile strength of C50 concrete.

It can be calculated from this that when the area is not equipped with anti-cracking steel bars and expansion joints, the reasonable maximum positive temperature gradient is 52.39 °C; when expansion joints are not installed, the reasonable maximum positive temperature gradient is 52.87 °C; when steel bars and expansion joints are set, they can meet the needs of the largest positive temperature gradient in the area.

## 5. Conclusions

Based on various local conditions of the Huaian County section of the Beijing–Zhangjiakou Railway, a numerical simulation model for the refined temperature field and temperature stress analysis of the CRTS I TDBBT system on the bridge has been established and its feasibility studied. The study revealed the following:

(1) The temperature gradient in the middle of the track slab is the largest; the maximum positive and negative temperature gradients are +62.32 and −31.02 °C, respectively.
(2) The middle of the surface of the track slab is prone to more cracks as longitudinal temperature stress is slightly larger than lateral temperature stress. At the edges of the sleeper, a 45° diagonal crack is likely to develop and propagate laterally. Deep cracks occur at the edge of the track slab as longitudinal temperature stress is much larger than lateral temperature stress.
(3) The longitudinal temperature stress of each measuring point along the track slab gradually increases, and the longitudinal temperature stress reaches the maximum at the mid-span of the track slab, where the increscent multiple can be up to 5.5 times.
(4) From the surface of the track slab to the bottom of the track slab, the lateral and longitudinal temperature stresses gradually reduce. The lateral and longitudinal stresses reduce by up to 4 times and 4.6 times, respectively.
(5) On increasing the anti-cracking reinforcement, the longitudinal stress of the track slab is reduced by at most 5.27%. Therefore, an increase in anti-cracking reinforcement helps to prevent cracking of the track slab due to a temperature load, but the effect is not significant.
(6) The third-order and second-order origin moment standard values of the temperature stress of the track on the traditional bridge are −3.104 and 2.814 MPa, respectively. After increasing the expansion joint, the origin moments reduce to −2.662 and 2.496 MPa, respectively, which leads to a decrease in the degree of temperature stress deviation and dispersion of the track, which enhances the resistance as compared to the traditional structure.

This research has carried out a study on the cracking mechanism of the track slab of the Huaian County section of the Beijing–Zhangjiakou Railway. It not only provides a theoretical basis for the design of the track slab of this section but also provides methods and ideas for research on the mechanism of variable-temperature cracking in other areas, which has engineering application significance.

## Figures and Tables

**Table 1:** Physical parameters of each structure of the CRTS I TDBBT.

Track Component	Physical Parameter	Numerical Value
Double-block sleeper	Density (kg/m^3^)	2.5 × 10^3^
	Thermal conductivity (W/m·k)	1.74
	Specific heat (J/kg·k)	9.2 × 10^2^
	Elastic modulus (MPa)	3.55 × 10^4^
	Coefficient of linear extensibility (1/°C)	1.0 × 10^−5^
	Poisson ratio	0.2
Track slab	Density (kg/m^3^)	2.5 × 10^3^
	Thermal conductivity (W/m·k)	1.74
	Specific heat (J/kg·k)	9.2 × 10^2^
	Elastic modulus (MPa)	3.2 × 10^4^
	Coefficient of linear extensibility (1/°C)	1.0 × 10^−5^
	Poisson ratio	0.2
Support layer	Density (kg/m^3^)	2.5 × 10^3^
	Thermal conductivity (W/m·k)	1.74
	Specific heat (J/kg·k)	9.2 × 10^2^
	Elastic modulus (MPa)	3.2 × 10^4^
	Coefficient of linear extensibility (1/°C)	1.0 × 10^−5^
	Poisson ratio	0.2
Reinforcement	Density (kg/m^3^)	7.85 × 10^3^
	Thermal conductivity (W/m·k)	58.2
	Specific heat (J/kg·k)	4.6 × 10^2^
	Elastic modulus (MPa)	2.06 × 10^5^
	Coefficient of linear extensibility (1/°C)	1.2 × 10^−4^
	Poisson ratio	0.3

## Data Availability

The data presented in this study are available on request from the corresponding author.
